# When Bystanders Skip CPR: Insights from a Retrospective Study

**DOI:** 10.3390/medicina62081524

**Published:** 2026-08-07

**Authors:** Giuseppe Stirparo, Elena Maria Ticozzi, Giulia Merigo, Aurora Magliocca, Annalisa Bodina, Gianluca Marconi, Gabriele Perotti, Giuseppe Ristagno, Carlo Signorelli, Marco Vinceti

**Affiliations:** 1 Agenzia Regionale Emergenza Urgenza (AREU), Via Campanini 6, 20124 Milan, Italy; 2 Department of Biomedical, Metabolic, and Neural Sciences, University of Modena and Reggio Emilia, 41125 Modena, Italy; 3 Department of Biomedical Sciences for Health, University of Milan, 20133 Milan, Italy; 4 Department of Pathophysiology and Transplantation, University of Milan, 20133 Milan, Italy; 5 Department of Anaesthesiology, Intensive Care and Emergency, Fondazione IRCCS Ca’ Granda Ospedale Maggiore Policlinico, 20122 Milan, Italy; 6 Faculty of Medicine, University Vita-Salute San Raffaele, 20132 Milan, Italy

**Keywords:** out-of-hospital cardiac arrest, gender, bystander CPR

## Abstract

*Background and Objectives*: Out-of-hospital cardiac arrest (OHCA) is one of the most clinically significant conditions and can lead to rapid death if cardiopulmonary resuscitation (CPR) or defibrillator use is not performed. Despite this, in approximately half of cases of witnessed OHCA, bystanders do not initiate resuscitation maneuvers. The factors underlying this phenomenon are varied but not yet fully understood. The aim of our analysis is to identify predictors that may indicate a higher likelihood of not performing resuscitation maneuvers. *Materials and Methods*: Data from cardiac arrests managed by the emergency medical system of the Lombardy region between 1 July 2024 and 30 June 2025 were analyzed. All missions involving cardiac arrests assisted only by lay bystanders were included in the analysis. *Results*: A total of 12,066 events were analyzed, of which only 4233 were assisted by laypeople. According to the logistic regression model, cardiac arrests occurring in urban settings (OR 0.60; 95% CI: 0.51–0.71), non-medical events (OR 0.44; 95% CI: 0.37–0.51), female patients (OR 0.80; 95% CI: 0.70–0.91), patients over 80 years old (OR 0.40; 95% CI: 0.35–0.45), and events occurring at home (OR 0.33; 95% CI: 0.28–0.39) were associated with a lower likelihood of performing chest compressions. Conversely, when the emergency service arrived within 15 min (OR 1.31; 95% CI: 1.14–1.51), the probability of receiving chest compressions increased. *Conclusions*: The presence of such a significant gap related to age and sex is noteworthy, prompting greater attention to the educational material presented during BLS-D courses. Moreover, these factors should be considered by the dispatch center when providing pre-arrival instructions to laypeople for initiating chest compressions. Specific communication training should be developed, and communication in these critical phases should be the subject of further study.

## 1. Introduction

Cardiac arrest (CA) is a critical medical emergency that can occur either in hospital (IHCA) or outside the hospital (OHCA). Despite advances in emergency care, OHCA remains associated with a high incidence and poor survival, with reported survival rates ranging from 3% to 10% [1]. Unlike IHCA, OHCA occurs in uncontrolled environments where immediate recognition and bystander intervention are crucial determinants of outcome. Due to its time-critical nature, delays in bystander-initiated resuscitation are closely linked with lower survival chances [2]. Therefore, early bystander CPR is a key component of basic life support (BLS-D) and should be initiated as soon as possible after OHCA occurrence [3]. In this context, early bystander CPR, together with public access defibrillation (PAD), represents a cornerstone of the chain of survival [4,5,6,7,8]. Thus, to ensure rapid bystander intervention, CPR training programs have become increasingly widespread, focusing on both theoretical and practical skill acquisition [4,9]. Furthermore, several systems have implemented first responder programs, enabling emergency dispatchers to alert trained lay responders to intervene before the arrival of Emergency Medical Services (EMS) [10].

However, despite widespread CPR training, bystander CPR is not initiated in all eligible OHCA cases [10,11]. To support lay rescuers, many EMS systems have implemented telephone-assisted CPR (TA-CPR), whereby dispatchers provide structured pre-arrival instructions (PAI) to facilitate early chest compressions [12]. Refining communication skills is also necessary to effectively guide lay rescuers [13,14,15,16,17].

Over the years, EMS systems have aimed to ensure a more rapid response to OHCA by promoting Basic Life and Support Defibrillation (BLS-D) training within the community [18]. Despite these efforts, bystander CPR is still not initiated in approximately half of OHCA cases [19]. The reasons underlying this gap remain multifactorial and incompletely understood. Investigating the underlying reasons for this high rate of inaction is critical for developing effective policies to improve CPR initiation and enhance patient survival outcomes.

The aim of this study was to identify demographic, clinical, organizational, and contextual factors associated with the provision of bystander CPR in patients with OHCA using data from the Lombardy AREU registry.

Although previous studies have explored barriers and facilitators associated with bystander CPR, the relative importance of these factors remains incompletely understood. In particular, demographic characteristics, previous training experiences, self-confidence, perceived competence, and psychological factors may all contribute to an individual’s readiness to intervene during a cardiac arrest emergency. A deeper understanding of these determinants is essential to design targeted educational strategies and public health interventions aimed at increasing bystander CPR rates [10].

Despite the growing dissemination of CPR training programs and community-based initiatives, bystander CPR rates remain suboptimal in many settings [19]. Identifying the demographic, clinical, organizational, and contextual factors associated with bystander CPR may help inform targeted educational strategies, optimize dispatcher-assisted CPR protocols, and guide public health interventions aimed at improving early resuscitation.

## 2. Materials and Methods

This registry-based study used data from the Agenzia Regionale Emergenza Urgenza (AREU), the regional agency responsible for coordinating the Emergency Medical Services (EMS) network in Lombardy, Italy, covering a catchment population of approximately 10 million inhabitants. This study was conducted in accordance with the principles of the Declaration of Helsinki. Data provided by the Agenzia Regionale Emergenza Urgenza (AREU) were fully anonymized before analysis. According to national regulations, Ethics Committee approval and informed consent were not required [20].

### 2.1. EMS in Lombardy Region

Lombardy is the largest and most populous region in Italy, with an area of 23,863 km^2^ and a population of 9.96 million inhabitants. In Lombardy, the pre-hospital emergency system is managed by the Regional Emergency Urgency Agency, AREU, which coordinates all emergency requests using wheeled vehicles or helicopters. In the event of an emergency, citizens can dial the European emergency number 112, which in the case of a medical condition transfers the call to the national 118 number for medical emergencies. A trained operator proceeds to assess the severity of the emergency by means of branched tree questions, assigning a severity code (white, green, yellow or red) to optimize the use of resources and vehicles, and the system was developed by an AREU internal board. The logistics of the rescue missions are coordinated by regional dispatch centers, which identify the most appropriate vehicle to be sent to the scene and the most appropriate hospital for the patient according to type and severity. All rescue missions are recorded in real time on the SAS-AREU portal, which contains logistical and medical details about each patient rescued by the emergency medical system. In the case of OHCA patients, MSB (basic life support vehicle) and an MSA (advanced cardiovascular vehicle) are sent to the scene. The vehicle has a GPS tracker and an automatic system which records the timing of rescue.

### 2.2. Data Registry and Analysis

Data regarding emergency rescue missions was retrieved from the SAS-AREU database. Emergency rescue missions involving out-of-hospital cardiac arrest (OHCA) were selected between 1 July 2024 and 30 June 2025.

Data management and preliminary cleaning were performed using Microsoft Excel to ensure completeness and consistency. Statistical analyses were conducted with Stata version 18. The following predictors were included in the model: age, gender, whether the event occurred within the municipality of a provincial capital (metropolitan area), rescue time (i.e., minutes elapsed between the emergency call and the arrival of the first emergency vehicle on scene), medical or non-medical causes of cardiac arrest, and location (i.e., home or public places). The normality of continuous variables was assessed using the Kolmogorov–Smirnov test. Data is reported as the median with the corresponding interquartile range (IQR). Differences in age distribution between sexes were evaluated using the Mann–Whitney U test. A multivariable logistic regression model was developed to examine the association between selected covariates and the outcome of interest (whether CPR was initiated by bystanders). The results are reported as odds ratios (ORs) with corresponding 95% confidence intervals (CIs).

## 3. Results

During the study period, the AREU system managed a total of 12,066 out-of-hospital cardiac arrests (OHCAs).

The demographic characteristics reveal notable sex-related differences: female patients were older than male patients (median age 83 years, IQR 74–89 vs. 74 years, IQR 61–84; *p* < 0.001), as also illustrated by the box-and-whisker plot of age distribution (Supplementary Material Figure S1). Additionally, the incidence per 100,000 inhabitants, stratified by age group and sex, increased progressively with age and remained consistently higher in males than in females, as show in Supplementary Material Graph S1. In fact, incidence remains very low and stable during early life and young adulthood for both sexes, then begins to rise from around middle age. From approximately 50–60 years onward, the increase becomes steeper, accelerating markedly after age 70. Across nearly all age groups, males exhibit consistently higher incidence rates than females, with the gap widening at older ages. In the oldest age groups (around 85–95 years), incidence rises sharply in both sexes, reaching the highest levels observed, with broader confidence intervals indicating greater variability in these age ranges. In Supplementary Material Figure S2, we show the distribution of out-of-hospital cardiac arrest (OHCA) cases according to the Utstein witness status classification. Unwitnessed arrests represented the largest subgroup (37.1%), followed by bystander-witnessed (35.1%) and EMS-witnessed events (7.9%).

**Figure 1:**
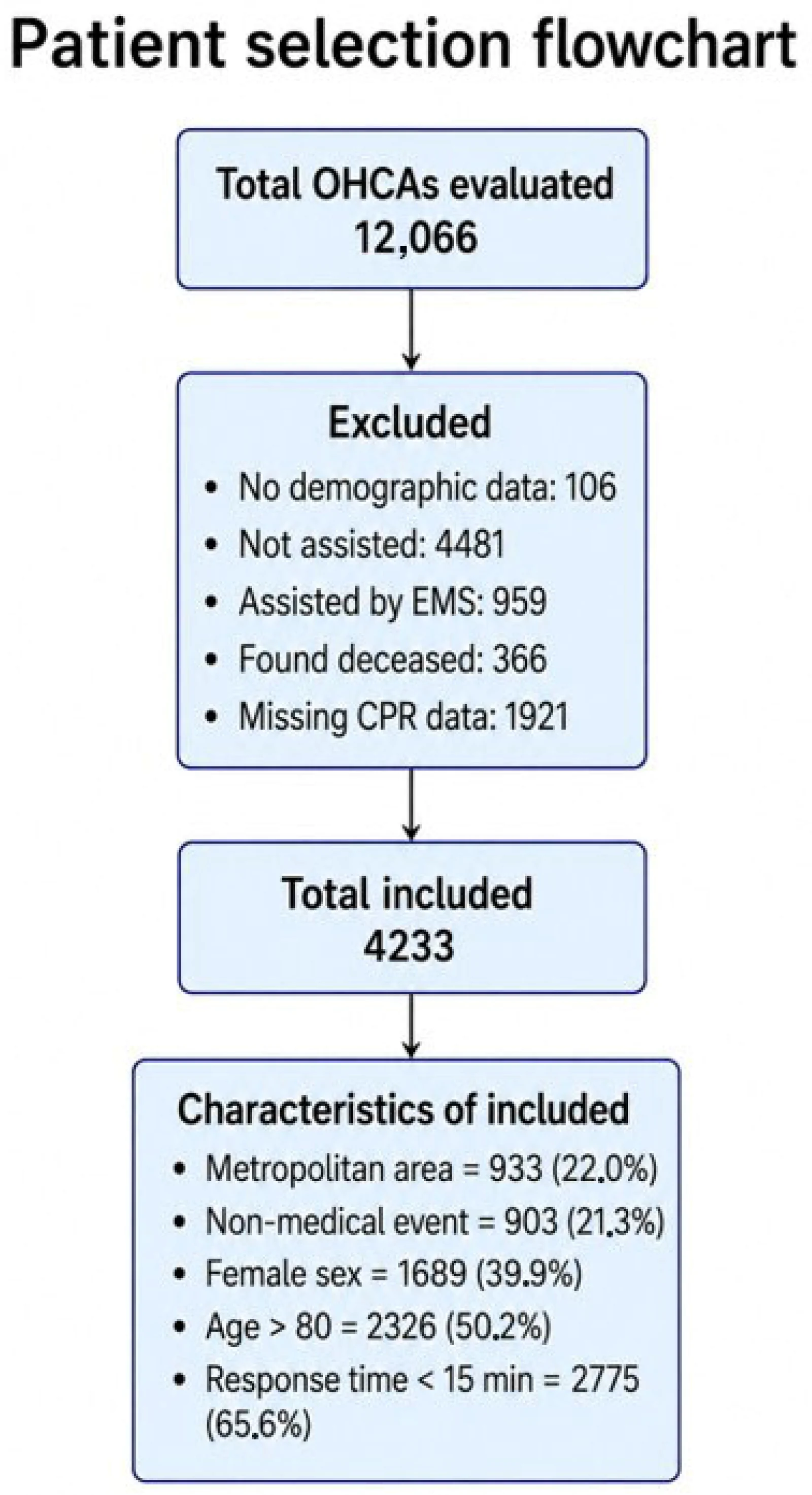
Patient selection flowchart.

Of these, 106 cases (0.89%) were excluded from the descriptive analysis due to missing data on age or gender. Figure 1 illustrates the patient selection process, in accordance with the study objectives. A total of 4233 patients that experienced a witnessed OHCA were included in the final analysis. Cases that were unwitnessed, that were witnessed by EMS personnel, or where the patient was found deceased were excluded.

Furthermore, 1921 patients were excluded due to missing data regarding the performance of CPR maneuvers. Among these, 784 (16.5% of the total number of cases) were female, and 1136 (15.7% of the total number of cases) were male, suggesting a low risk of sex-based selection bias.

Of the 4233 patients included, 2188 (51.6%) received bystander CPR. Regarding clinical outcomes, 517 (12.2%) achieved return of spontaneous circulation (ROSC) before hospital arrival, 2556 (60.4%) died on the scene and 1160 (27.4%) were transported to the hospital. The median arrival time for EMS vehicles was 13 min [IQR: 10–16], and the median time to complete the mission was 55 min [IQR: 44–65]. The median age of the study population was 79 years [IQR: 66–87].

**Figure 2:**
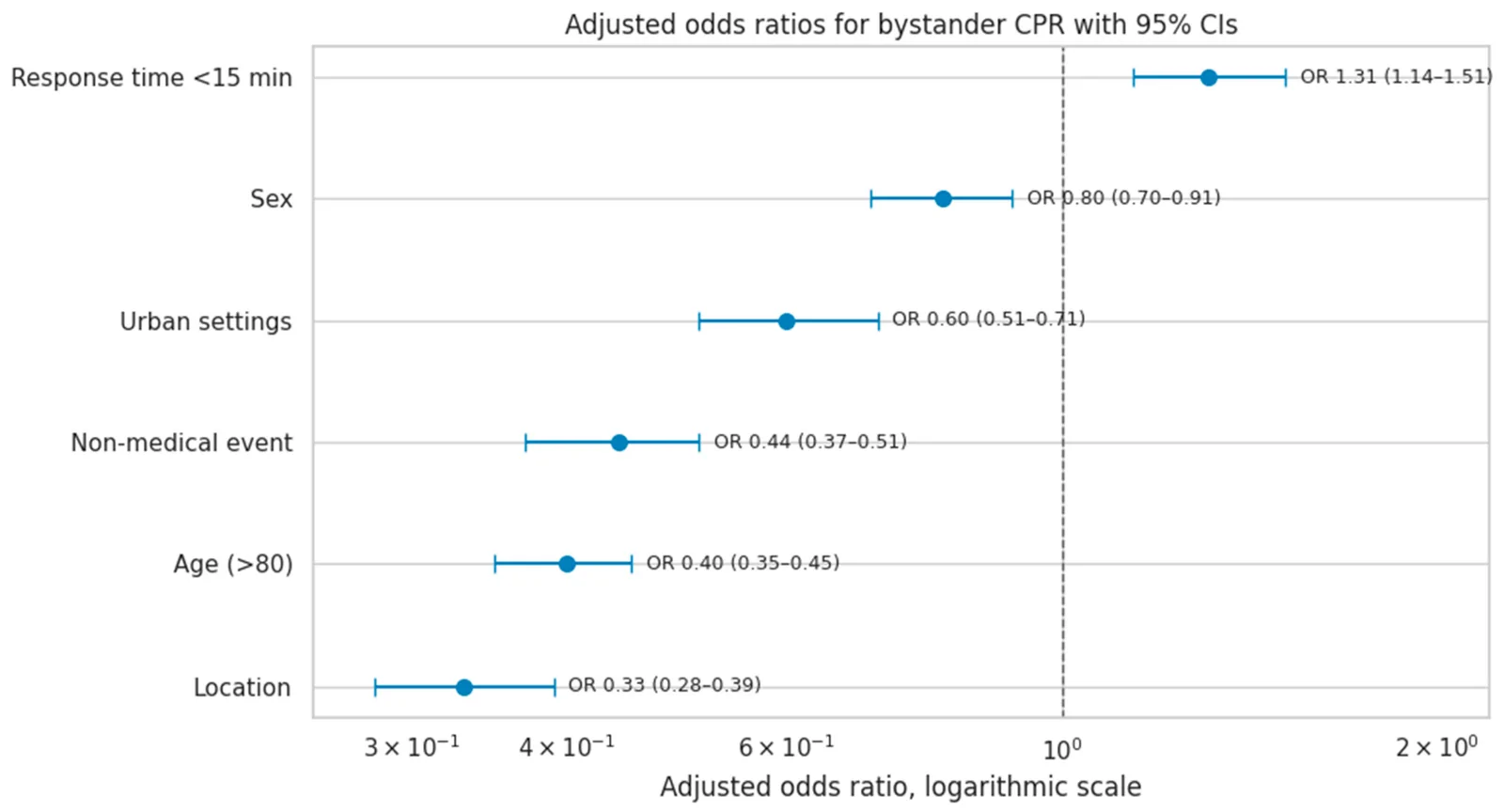
Logistic regression of CPR outcomes based on bystanders.

Figure 2 illustrates the results of the multivariate logistic regression model regarding the factors associated with bystander CPR. The analysis indicates that OHCA occurring in urban settings (OR: 0.60; 95% CI: 0.51–0.71), non-medical events (OR: 0.44; 95% CI: 0.37–0.51), female sex (OR: 0.80; 95% CI: 0.70–0.91), patient age over 80 years old (OR: 0.40; 95% CI: 0.35–0.45), and location defined as a residential setting (OR: 0.33; 95% CI: 0.28–0.39) are all independently associated with a decreased likelihood of bystander intervention. Conversely, an EMS response time of less than 15 min (OR 1.31; 95% CI: 1.14–1.51) is positively associated with a higher probability of bystander CPR.

**Figure 3:**
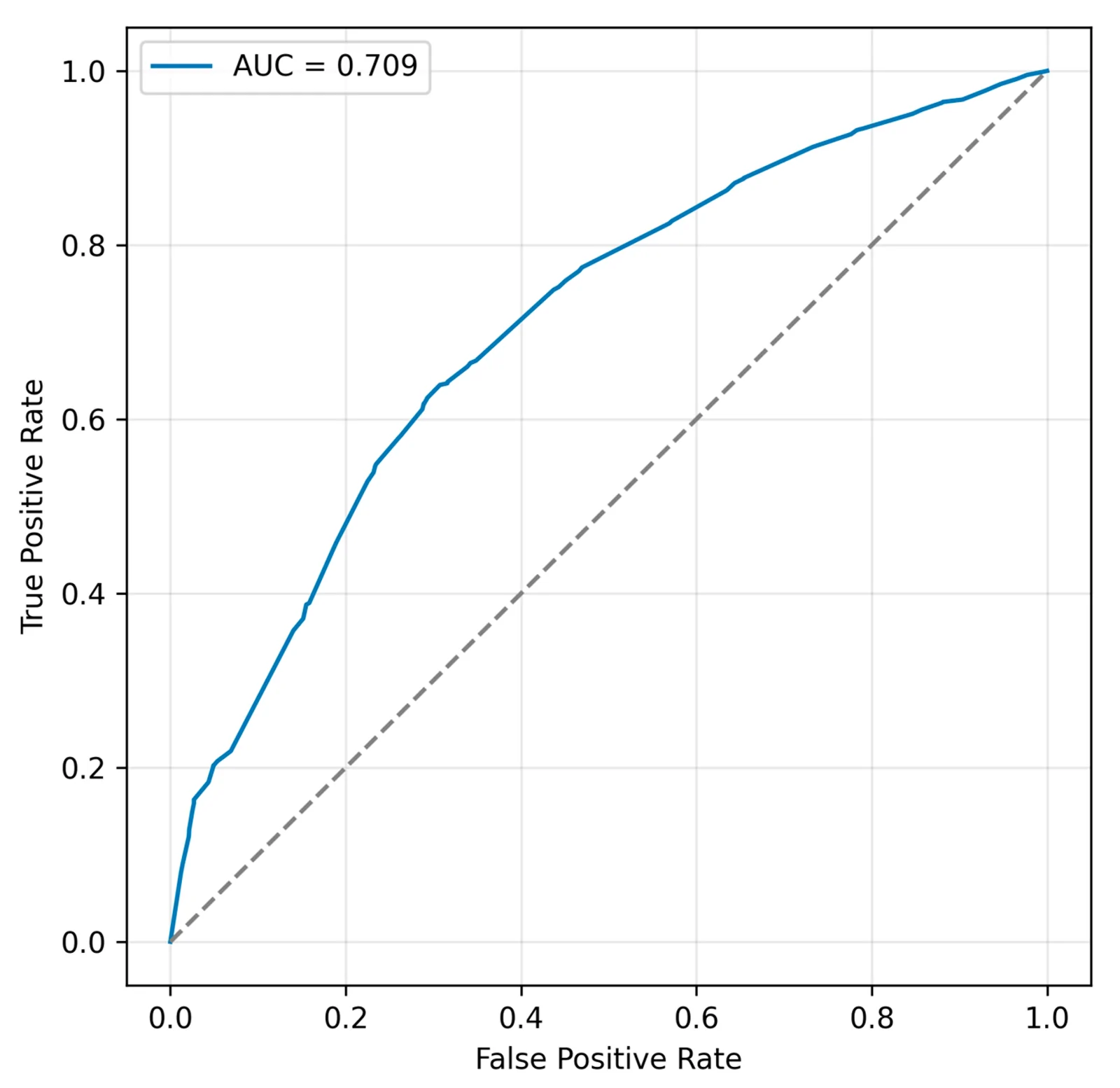
ROC curve of multivariable logistic regression model’s CPR outcomes.

The discriminatory performance of the multivariable logistic regression model was further evaluated using receiver operating characteristic (ROC) curve analysis. The model achieved an area under the ROC curve (AUC) of 0.709, indicating an acceptable ability to discriminate between patients with and without the outcome of interest, as shown in Figure 3.

## 4. Discussion

The present study identified several organizational, contextual, and demographic factors associated with the likelihood of bystander-initiated CPR in OHCA. Specifically, shorter EMS response times were associated with higher intervention rates, whereas female sex, advanced age, non-medical etiology, and a residential setting were independently associated with a lower probability of receiving bystander CPR.

Our study analyzed 12,066 cases of OHCAs, with an incidence rate consistent with previously published data [1,21,22]. The proportion of OHCAs witnessed by bystanders (35%) was slightly lower than that reported in other European cohorts [1]. Conversely, the rate of bystander-initiated CPR (51.6%) was comparable to the average European figures reported in the EuReCa study [1]. Furthermore, the distribution of non-medical events, residential arrest patients aged over 80 years, and female patients was aligned with the existing literature [22]. These baseline demographic and clinical characteristics supported the representativeness of our study population, providing a robust foundation for addressing our primary research objectives.

In Supplementary Material Figure S2, the distribution of out-of-hospital cardiac arrest (OHCA) cases according to the Utstein witness status classification. Unwitnessed arrests represented the largest subgroup (37.1%), followed by bystander-witnessed (35.1%) and EMS-witnessed events (7.9%). As expected, witnessed arrests were associated with more favorable pre-hospital outcomes than unwitnessed events. In particular, EMS-witnessed arrests showed the highest rate of return of spontaneous circulation (20.3%), while bystander-witnessed arrests had the highest proportion of patients transported to the emergency department (42.5%). Conversely, unwitnessed arrests were characterized by the lowest ROSC rate (4.0%) and the highest proportion of deaths occurring on scene (78.0%). These findings are consistent with the established prognostic value of witnessed status and emphasize the critical role of early recognition and timely initiation of the chain of survival in improving OHCA outcomes.

An additional factor that may have influenced the observed outcomes is the organizational structure of the Lombardy EMS system. MSBs (basic life supper vehicle) are frequently staffed by trained volunteers who, according to regional protocols, are not authorized to certify death. Consequent an MSA (advanced cardiovascular vehicle) including a physician may still be dispatched solely to perform the formal confirmation of death. These events are therefore recorded within the EMS response despite no active resuscitation attempt being undertaken. As a result, the inclusion of such cases may contribute to reducing the ROSC and survival rates compared with registries in which patients with obvious signs of death or no attempted resuscitation are excluded before analysis.

The multivariate regression analysis identified several factors associated with the likelihood of bystander-initiated CPR in this regional OHCA cohort. OHCA patients in urban settings were less likely to receive bystander CPR compared to rural areas (OR: 0.60; 95% CI: 0.51–0.71). This association may have reflected the inherent complexity of urban environments, where the presence of numerous bystanders did not necessarily translate into immediate intervention. Furthermore, non-medical events (i.e., trauma-related) were associated with a lower likelihood of CPR (OR: 0.44; 95% CI: 0.37–0.51), potentially because such scenarios were perceived as more complex and technically difficult than medical emergencies. Regarding gender disparities, female patients faced lower odds of receiving bystander CPR (OR: 0.80; 95% CI: 0.70–0.91), a finding consistent with the existing literature [23]. This disparity may have been related to cultural factors, including the misconception that women were at lower risk of OHCA and a higher level of hesitation regarding physical contact during resuscitation [24,25,26,27]. Finally, in line with previous studies, younger age was a significant predictor of receiving CPR. This may have reflected a combination of better-witnessed events, a more rapid activation of EMS, and a societal tendency toward more prompt resuscitation efforts for younger individuals [28].

Patients over the age of 80 were also less likely to receive bystander CPR (OR: 0.40; 95% CI: 0.35–0.45). In such cases, one possible explanation is that severe comorbidities and perceptions regarding prognosis may have contributed to hesitation among bystanders. The present findings underscored the importance of ensuring that bystanders did not subjectively determine the appropriateness of resuscitation. These findings reinforce the importance of encouraging bystanders to follow dispatcher instructions, as decisions regarding the initiation and termination of resuscitation ultimately rest with trained healthcare professionals. Similarly, OHCAs occurring in a residential setting were associated with a reduced likelihood of receiving bystander CPR (OR: 0.33; 95% CI: 0.28–0.39). This may have been explained by the fact that family members, who are often the primary witnesses, may have felt unprepared or emotionally overwhelmed. In contrast, public or workplace settings may have been more likely to involve individuals with previous CPR training or easier access to automated external defibrillators (AEDs) [9,10,11,12,13,14,15,16,17,18,19,20,21,22,23,24,25,26,27,28,29,30]. This finding was particularly noteworthy because family members might be expected to intervene more readily owing to their emotional bond with the victim. Consequently, these findings supported the development of specific communication protocols for emergency dispatchers to help family members overcome emotional barriers and effectively guide them through resuscitation during PAI calls.

Evaluating EMS response time, a response interval of less than 15 min was associated with a higher likelihood of bystander CPR (OR 1.31; 95% CI: 1.14–1.51). Given the observational nature of this study, this association should not be interpreted as causal. Rather, shorter EMS response times may represent a marker of the earlier recognition of OHCA, a more rapid activation of the emergency response system, or other unmeasured characteristics of the emergency event that were also associated with a greater likelihood of bystander intervention.

In Figure 3, the ROC curve analysis shows that the proposed logistic regression model had an acceptable discriminative ability, with an AUC of 0.709. Although this level of performance is not indicative of excellent prediction, it suggests that the model is able to distinguish patients at higher risk from those at lower risk with reasonable accuracy. The moderate AUC also indicates that additional predictors, including clinical, biochemical, and imaging variables, may further improve model performance. Future studies involving larger, multicenter cohorts and external validation are needed to refine the model and confirm its applicability in different clinical settings.

The factors influencing OHCA survival remained multifaceted. Women, in particular, exhibited higher mortality rates compared to men, even when pre-hospital care was standardized [22]. Reducing this gender disparity should be considered a priority for future educational initiatives, particularly given evidence that women may have had lower physiological tolerance to coronary events. To address this issue, one potential strategy may be the greater integration of female mannequins into BLS-D training programs, as they are currently underrepresented in most training programs [31]. Furthermore, training programs could place greater emphasis on the initiation of chest compressions in female patients, capitalizing shared opportunities with students [32]. Education remains a critical vehicle for awareness, as women were less likely to initiate EMS activation, and their cardiac events were often recognized later due to the presence of atypical signs and symptoms [33,34]. Finally, developing targeted communication protocols for dispatch center operators is highly recommended [35,36].

The considerable improvements achieved in recent decades in out-of-hospital cardiac arrest survival were closely linked to the widespread dissemination of CPR training programs and the growing involvement of lay rescuers in the chain of survival. Nevertheless, despite the increasing number of trained individuals within the community, bystander CPR rates remained suboptimal in many settings, highlighting the need for continued efforts to better understand the factors influencing willingness to intervene. Studies such as the present one are therefore essential to identify factors associated with bystander CPR and to generate hypotheses regarding the barriers and facilitators that may influence the translation of acquired knowledge into real-life action. The findings could support the development of more effective educational strategies, tailored training programs, and targeted public health initiatives aimed at strengthening confidence and readiness among potential rescuers.

Within this regional cohort, these findings suggested that the barriers to bystanders performing CPR extend beyond the mere availability of training programs. It was unlikely that many of the factors identified in our analysis—including advanced age, female gender, residential location and traumatic etiology—could be addressed solely through conventional CPR courses. This highlighted the need for a broader approach that combines technical training with interventions aimed at improving the recognition of cardiac arrest, reducing hesitation and increasing confidence in emergency situations. Particular attention should be paid to family members and those caring for people at higher cardiovascular risk, as they were the most likely bystanders to witness a cardiac arrest occurring at home. Similarly, emergency call centers could play a key role in supporting bystanders by providing structured instructions before the arrival of emergency services, helping to alleviate uncertainty and emotional distress during the early stages of resuscitation. Future initiatives should focus on identifying the most effective strategies for putting CPR knowledge into practice when a real emergency occurs.

Although substantial improvements in survival following out-of-hospital cardiac arrest have been reported in recent decades, the findings of this study indicated that there is still considerable room for improvement. Almost one in two patients did not receive CPR before the arrival of the emergency medical services, despite the well-established link between early chest compressions and survival. This finding was particularly significant given the considerable efforts devoted to community CPR training and public awareness campaigns. The persistence of disparities based on patient characteristics and the circumstance of the cardiac arrest suggested that barriers to intervention still exist and may not be adequately addressed by current training models. Understanding the possible factors associated with the absence of bystander CPR in regional EMS systems, as well as the behavioral and contextual determinants underlying these associations, may help guide future interventions. Further research is needed to explore the behavioral, social, and contextual factors underlying the associations identified in this study and to evaluate interventions specifically designed to reduce these barriers. Improving bystander readiness to act remains a key objective for increasing CPR rates and improving survival following OHCA.

This study has several limitations. First, its retrospective observational design, based on a local registry, is inherently subject to potential selection bias and unmeasured confounding. Although data were collected prospectively, missing or incomplete variables may have introduced residual bias into the analyses. Second, the absence of long-term outcome data beyond six months, including quality of life and functional recovery, precludes a comprehensive assessment of patient-centered outcomes. Third, this study was based exclusively on data from the Lombardy AREU registry. Therefore, differences in emergency medical service organization, dispatcher-assisted CPR protocols, public CPR training, and population characteristics may limit the generalizability of our findings to other regions or healthcare systems. Finally, due to the observational nature of this study, causality cannot be inferred from the observed associations.

## 5. Conclusions

This registry-based study from Lombardy demonstrates that several organizational, contextual, and demographic factors significantly influence the likelihood of bystander-initiated CPR. Shorter EMS response times were positively associated with higher intervention rates, whereas female sex, advanced age, non-medical etiology, and a residential setting were associated with a lower probability of receiving bystander assistance. These findings highlight the urgent need for targeted public health strategies to reduce disparities in bystander intervention and strengthen community response to OHCA. Expanding CPR training and supporting bystanders through effective dispatcher-assisted CPR may contribute to improving early resuscitation and patient outcomes.

## Data Availability

The data that support the findings of this study are available from the corresponding author upon reasonable request. Data are not publicly available because they belong to the AREU registry and are subject to institutional data governance policies.

## Supplementary Materials

The following supporting information can be downloaded at https://www.mdpi.com/article/10.3390/medicina62081524/s1, Figure S1: Demographic characteristics. Graph S1: Incidence per 100,000 inhabitants by age group and sex. Figure S2: OHCA group by Utstein classification.
