# Supplementary material for: When Bystanders Skip CPR: Insights from a Retrospective Study
Source: Medicina (Kaunas). 2026 Aug 7;62(8):1524. doi: 10.3390/medicina62081524 (PMC13515388; doi:10.3390/medicina62081524)

Supplementary S1 - demographic characteristics:

Female patients were older than male patients (median age 83 years, IQR 74–89 vs. 74 years, IQR 61–84; Mann–Whitney U test, U = 21,307,771.5, Z = 30.81, $p < 0.001$).

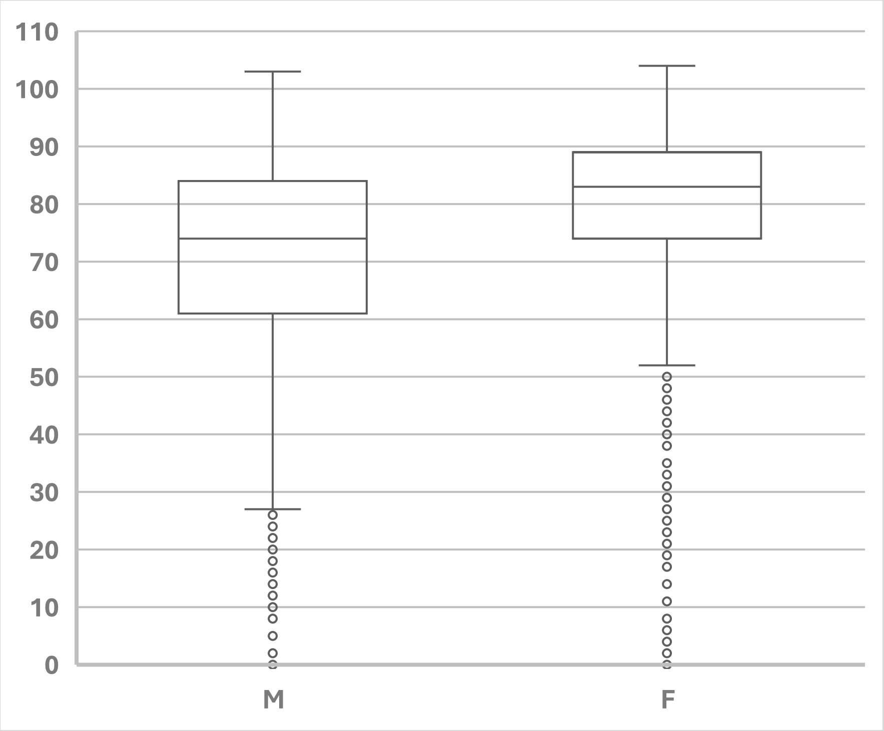

Figure S1: Box-and-whisker plot showing the age distribution by sex.

The incidence per 100,000 inhabitants by age group and sex. Incidence increased progressively with age and was consistently higher among males compared with females.

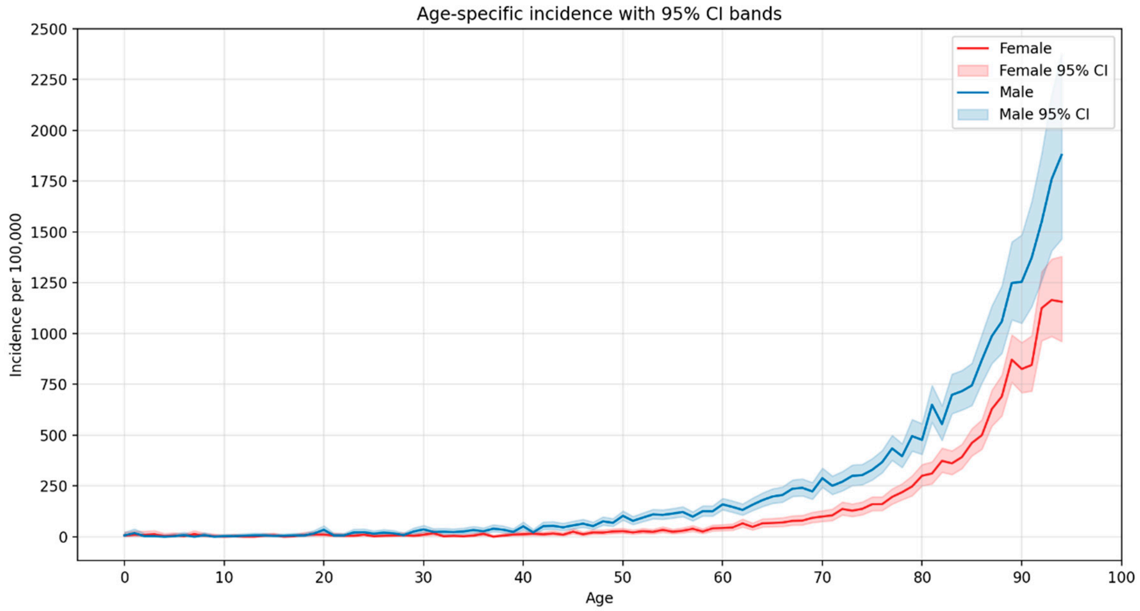

Graph S1: incidence per 100,000 inhabitants by age group and sex

Supplementary S2: Utstein Classification

Figure S2: OHCA group by Utstein Classification

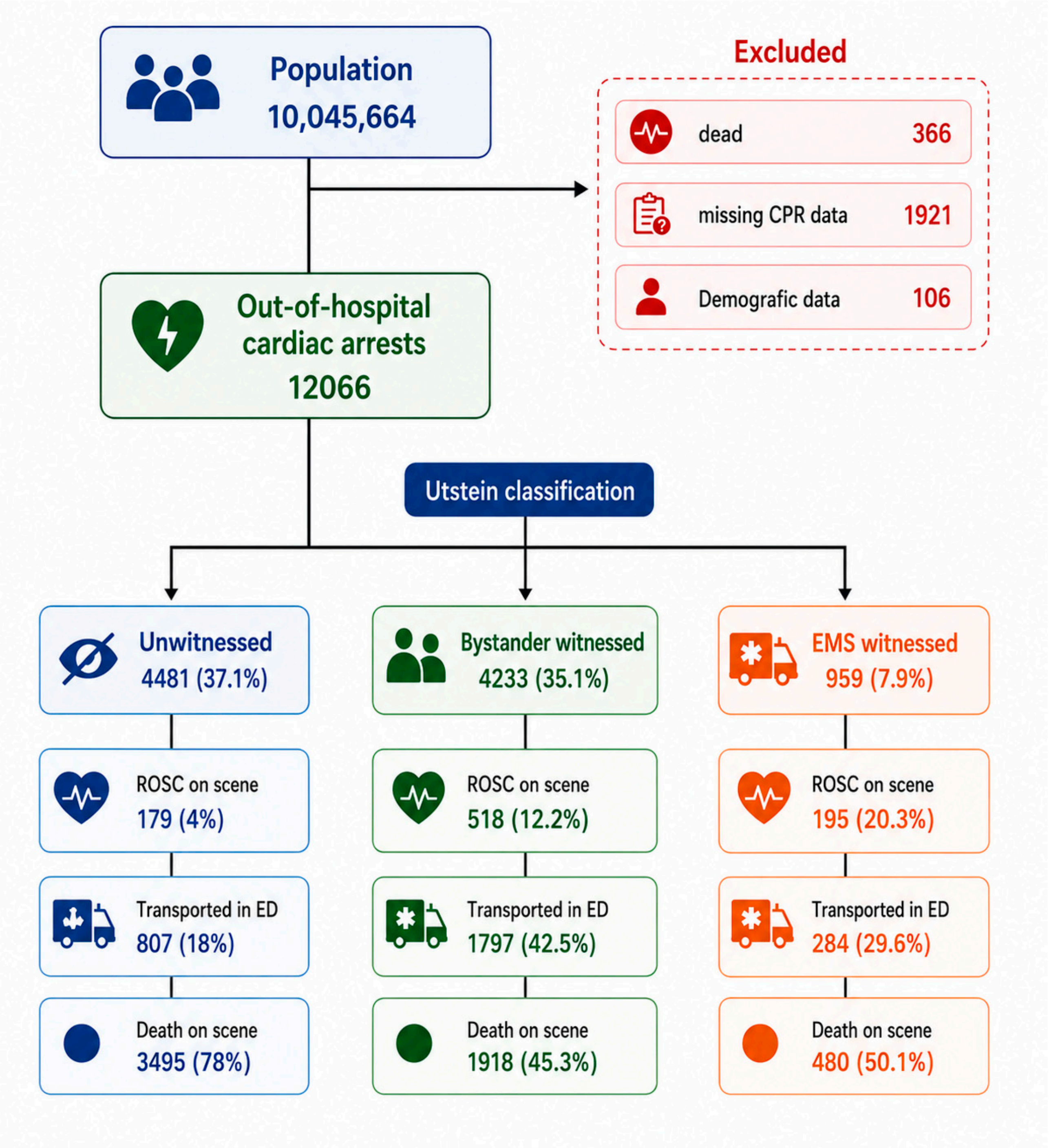

Supplement: Supplementary file 1 [file medicina-62-01524-s001.zip › medicina-4423163-supplementary.pdf]
